# Cost of illness of ischemic heart disease in Japan: a time trend and future projections

**DOI:** 10.1186/s12199-018-0708-1

**Published:** 2018-05-24

**Authors:** Toshiharu Gochi, Kunichika Matsumoto, Rebeka Amin, Takefumi Kitazawa, Kanako Seto, Tomonori Hasegawa

**Affiliations:** 1 Department of Social Medicine, Toho University Graduate School of Medicine, 5-21-16 Omori-nishi, Ota-ku, Tokyo, 143-8540 Japan; 2 Department of Social Medicine, Toho University School of Medicine, 5-21-16 Omori-nishi, Ota-ku, Tokyo, 143-8540 Japan

**Keywords:** Cost of illness, Ischemic heart disease, Medical economics, Health policy, Aging

## Abstract

**Background:**

Ischemic heart disease (IHD/ICD10: I20-I25) is the second leading cause of deaths in Japan and accounts for 40% of deaths due to heart diseases. This study aimed to calculate the economic burden of IHD using the cost of illness (COI) method and to identify key factors that drive the change of the economic burden of IHD.

**Methods:**

We calculated the cost of illness (COI) every 3 years from 1996 to 2014 using governmental statistics. We then predicted the COI for every 3 years starting from 2017 up to 2029 using the fixed and variable model estimations. Only the estimated future population was used as a variable in the fixed model estimation. By contrast, variable model estimation considered the time trend of health-related indicators over the past 18 years. We derived the COI from the sum of direct and indirect costs (morbidity and mortality).

**Results:**

The past estimation of COI slightly increased from 1493.8 billion yen in 1996 to 1708.3 billion yen in 2014. Future forecasts indicated that it would decrease from 1619.0 billion yen in 2017 to 1220.5 billion yen in 2029.

**Conclusion:**

The past estimation showed that the COI of IHD increased; in the mixed model, the COI was predicted to decrease with the continuing trend of health-related indicators. The COI of IHD in the future projection showed that, although the average age of death increased by social aging, the influence of the number of deaths and mortality cost decreased.

## Background

Ischemic heart disease (IHD/ICD10: I20-I25) is the second leading cause of death in Japan and accounts for 40% of deaths due to heart diseases. Recently, the number of deaths (NDy) by IHD has been almost stable; however, the average age of death has increased [1]. In 2014, 73,862 individuals (men 41,902; women 31,960) died because of IHD in Japan.

The influence of the social burden in IHD is expected to change with aging of the Japanese population, because IHD has a high mortality rate in the older age group.

The objectives of this study were to estimate the social burden of IHD using the cost of illness (COI) method and to predict future changes. The COI method has been widely used since the 1960s after being introduced by Rice et al. as a method that can economically evaluate disease burden [2–12]. As the disease was already studied by another group, the results of which were similar to those evaluated above, the calculation method used in this study is similar to that used in our previous studies [13–19]. We estimated the economic burden of major forms of cancer from 1996 and projected the future economic burden [13–17]. These analyses showed that social aging and increase in the average age of death had an impact on the decreased economic burden by devaluing human capital. However, there were no previous studies concerning the economic burden of IHD, despite it being a significant cause of deaths. This study estimated the economic burden of IHD on time trends and determined the effects of change and the aging of the burden that IHD poses on society by surveying the change predicted in the future.

## Methods

This COI study used a top–down method to estimate resource consumption using governmental aggregated data. COI was estimated using the incidence-based approach. The calculation method used in this study was the same as that in our previous studies investigating COI [13–19]. COI was estimated as the sum of direct cost (DC) and indirect cost (IC), with IC divided into morbidity cost (MbC) and mortality cost (MtC) as follows:

$$ \mathrm{COI}=\mathrm{DC}+\mathrm{MbC}+\mathrm{MtC}. $$

DC is a medical cost directly related to the disease and includes costs associated with treatment, hospitalization, laboratory investigations, and drugs. Furthermore, DC comprises hospitalization cost (HC) and outpatient cost (OC). DC was calculated using the following equation:

$$ \mathrm{DC}=\mathrm{HC}+\mathrm{OC}=\mathrm{iCd}\times \mathrm{THD}+\mathrm{oCd}\times \mathrm{TOVy}. $$

HC was determined by multiplying the inpatient cost per day (iCd) with the total person-days of hospitalization (THD). OC was determined by multiplying the OC per day (oCd) with the total person-days of outpatient visits (TOVy).

Here, we calculated the annual medical costs from the total medical expenses using the “Survey of National Medical Care Insurance Services” [20].

MbC is the opportunity cost lost resulting from hospitalization and visit to hospitals. We calculated MbC using the following equation:

$$ \mathrm{MbC}=\mathrm{TOVy}\times \mathrm{LVd}/2+\mathrm{THD}\times \mathrm{LVd}. $$

LVd is the 1-day labor value per person. We calculated TOVy and THD in 5-year age groups based on the “Patient Survey” [21] conducted every 3 years by the Japanese government. We determined the labor values in the 5-year age groups based on data from “Basic Survey on Wage Structure” [22], “Labor Force Survey” [23], “Estimates of Monetary Valuation of Unpaid Work” and “Evaluations of Domestic Labor” [24]. We determined MbC by assuming a 1-day labor value loss per day at the hospital and a half-day labor value loss per outpatient visit. We calculated LVd and THD as follows:

$$ \mathrm{LVd}=\left(\mathrm{Iy}+\mathrm{ULVy}\right)/365 $$

$$ \mathrm{THD}=\mathrm{HPy}\times \mathrm{ALOS} $$

where Iy is the annual income per person, ULVy is the annual monetary valuation of unpaid work per person, HPy is the annual number of hospitalized patients, and ALOS is the average length of hospital stay. MtC is measured as the loss of human capital (human capital method), which was calculated using the following equation:

$$ \mathrm{MtC}=\mathrm{NDy}\times \mathrm{LVl} $$

where NDy is the number of deaths and LVl is the lifetime labor value per person. We calculated NDy of IHD for each 5-year age group based on “Vital Statistics of the Ministry of Health, Labor and Welfare” [1]. We calculated LVl by summing the present value of the potential future income should the patient survives. By estimating and projecting the COI values, the rate of contribution of DC, MbC, and MtC to the overall COI variation was calculated as follows:

$$ \frac{{\mathrm{Cost}}_t^i-{\mathrm{Cost}}_0^i}{{\mathrm{COI}}_t-{\mathrm{COI}}_0} $$

${\mathrm{Cost}}_t^i$: DC, MbC, and MtC costs at year *t*

${\mathrm{Cost}}_0^i$: DC, MbC, and MtC costs at the baseline year

COI_*t*_: COI at year *t*

COI_0_: COI at the baseline year

With regard to the potential future labor value, we conducted a sensitivity analysis for the discount rate. Thus, the base case discount rate was 3%, and our analyses included a discount rate of 0–5%.

We used data from “Population Estimates” [25] published by the Ministry of Internal Affairs and Communications for 1996–2014. The data for 2017–2029 were drawn from the “Population Statistics of Japan” [26] published by the National Institute of Population and Social Security Research. We used the projection based on medium fertility and medium mortality assumptions. The year 2014 was selected as the benchmark for the 1-day labor value by sex and 5-year age groups. Two methods were used for the future projection of MbC and MtC: the “fixed” and “variable” methods. We estimated the rate of change of four health-related indicators over the past 18 years, namely, the mortality rate, per capita outpatient visits, per capita outpatient hospitalizations, and ALOS. The “fixed” method fixed the health-related indicators of each age group at the 2014 level and changed only the future population and age structure. The “variable” method accounted for changes in health-related indicators in addition to population and age structure. Regarding variable model estimation, we estimated linear (linear model) and logarithmic regression for upward trends or exponential regression for downward trends (exponential/logarithmic model) by changing health-related indicators. We then assessed the mixed model estimation by adopting a higher approximation of the decision coefficient every 5 years. Theoretically, the mixed model was the most valid, and the fixed model could be considered the reference. With regard to ALOS, we adopted the value of 2.7 days from Norway, which was obtained from OECD Health Data 2014 (Statistics and Indicators), because it was the lowest value among all 34 OECD countries (2014) for IHD. This “2.7 days” was applied for age groups that were calculated to be < 2.7 days from the estimated value of 2017–2029.

The study protocol was approved by the Ethics Committee of the Toho University School of Medicine (reference number: A16019).

## Results

### Past estimation of COI

COI increased by an average of 14.4% from 1996 to 2014. DC and MtC increased by 18.1 and 15.0%, respectively, whereas MbC decreased by 18.8% from 1996 to 2014. The contribution rates of DC, MbC, and MtC were 42.7, − 6.1, and 63.4%, respectively. NDy increased by 2.8%, but the proportion of NDy in persons aged ≥ 65 years and the average age of death increased from 1996 to 2014. As for the mortality rate, decreases were observed in almost all age groups. The proportion of MtC of persons aged ≥ 65 years, MtC per capita, which was calculated by dividing MtC by NDy, increased by 11.9% from 1996 to 2014 (Table 1).

**Table 1:** The time trend of cost of illness (COI) of ischemic heart disease

Item	1996	1999	2002	2005	2008	2011	2014
Population (thousand person)	125,864	126,686	127,435	127,768	127,692	127,799	126,949
[% of persons aged ≥ 65 years]	15.1%	16.7%	18.5%	20.2%	22.1%	23.3%	26.1%
Number of deaths (person)	71,858	73,894	71,507	76,474	76,564	77,168	73,862
[% of persons aged ≥ 65 years]	83.5%	83.7%	83.6%	84.3%	85.4%	85.5%	87.1%
Average age of death (year)	75.6	75.7	75.8	76.3	76.8	77.1	77.5
[Men]	72.4	72.3	72.4	72.9	73.6	74.1	74.6
[Women]	79.2	79.6	79.9	80.4	80.8	81.1	81.4
Direct cost (billion yen)	507.3	534.1	527.6	515.9	578.9	561.4	598.9
Morbidity cost (billion yen)	82.3	117.2	106.9	96.0	85.1	75.4	69.3
Mortality cost (billion yen)	904.2	912.1	1060.0	1057.0	1054.5	1150.9	1040.1
[% of persons aged ≥ 65 years]	35.7%	35.2%	42.1%	42.9%	45.2%	49.8%	50.0%
Mortality cost per person (million yen)	12.6	12.3	14.8	13.8	13.8	14.9	14.1
COI (billion yen)	1493.8	1563.4	1694.5	1668.9	1718.5	1787.6	1708.3

Source of population: Ministry of Internal Affairs and Communications “Population Estimates.” Average age of death: calculated according to the number of deaths, sex, and age (5-years old age grade), cause of death in “Vital Statistics”

Regarding the calculation of DC from 1996 to 2014, THD and TOVy decreased, whereas iCd and oCd increased. The rates of change for THD and TOVy were − 66.1 and − 44.0%, respectively, whereas those for iCd and oCd were 440.9 and 143.1%, respectively.

### Future projection of COI

#### Fixed model

**Fig. 1:**
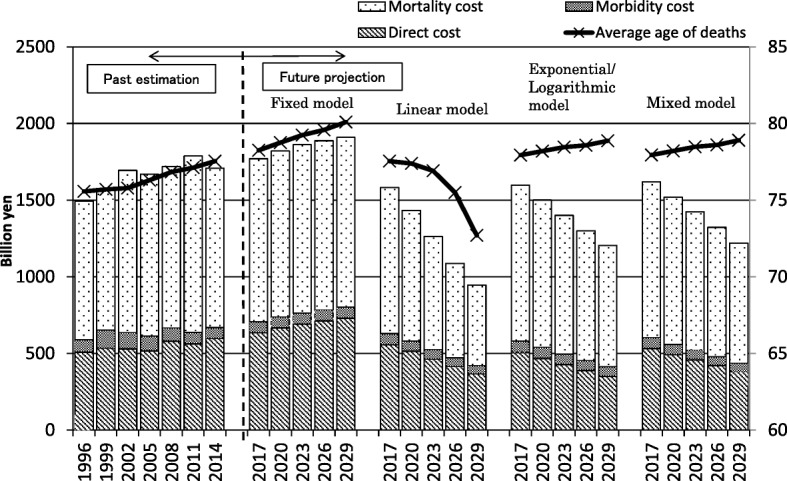
Cost of illness (COI) projection with cost elements and projection of average age of death

COI tended to increase from 2017 to 2026. It was projected to increase by 11.8% from 2014 to 2029. DC, MbC, and MtC also increased in the same period. The contribution rates of DC, MbC, and MtC were 64.5, 1.6, and 21.9% from 2014 to 2029, respectively. NDy was also predicted to increase, and the increase rate was 36.6% from 2014 to 2029. The proportion of NDy in persons aged ≥ 65 years increased, accounting for 90.7% of NDy due to IHD in 2029, and the average age of death was also predicted to increase from 77.5 years in 2014 to 80.1 years in 2029. The proportion of MtC of persons aged ≥ 65 years was predicted to be 55.6% in 2029, but the MtC per capita was projected to decrease by 22.0% from 2014 to 2029 (Table 2, Fig. 1).

**Table 2:** Future prediction of cost of illness (COI) of ischemic heart disease

	Item	2017	2020	2023	2026	2029
	Estimated population (thousand person)	125,739	124,100	122,122	119,891	117,465
	[% of persons aged ≥ 65 years]	28.0%	29.1%	29.8%	30.5%	31.2%
Fixed model	Number of deaths (person)	80,569	86,096	91,553	95,522	100,894
	[% of persons aged ≥ 65 years]	88.8%	89.6%	90.0%	90.3%	90.7%
	Average age of death (year)	78.3	78.7	79.2	79.6	80.1
	Direct cost (billion yen)	635.7	665.0	690.9	710.2	728.9
	Morbidity cost (billion yen)	70.5	71.8	72.4	72.6	72.6
	Mortality cost (billion yen)	1064.0	1083.7	1098.2	1104.7	1108.2
	[% of persons aged ≥ 65 years]	53.1%	54.0%	54.5%	54.8%	55.6%
	Mortality cost per person (million yen)	13.2	12.6	12.0	11.6	11.0
	COI (billion yen)	1770.2	1820.5	1861.5	1887.5	1909.7
Linear model	Number of deaths (person)	66,367	57,789	47,249	34,512	23,860
	[% of persons aged ≥ 65 years]	86.5%	85.3%	82.6%	77.1%	68.5%
	Average age of death (year)	77.5	77.4	76.9	75.5	72.7
	Direct cost (billion yen)	556.4	514.1	462.7	415.4	367.0
	Morbidity cost (billion yen)	73.0	67.4	61.6	56.6	52.3
	Mortality cost (billion yen)	953.6	850.8	737.9	615.5	525.9
	[% of persons aged ≥ 65 years]	47.3%	43.5%	37.3%	28.5%	21.8%
	Mortality cost per person (million yen)	14.4	14.7	15.6	17.8	22.0
	COI (billion yen)	1583.0	1432.4	1262.2	1087.5	945.3
Exponential/logarithm model	Number of deaths (person)	73,782	71,108	68,157	64,163	61,072
	[% of persons aged ≥ 65 years]	87.8%	87.8%	87.6%	87.1%	86.8%
	Average age of death (year)	77.9	78.2	78.5	78.6	78.9
	Direct cost (billion yen)	504.1	465.4	425.2	386.1	351.2
	Morbidity cost (billion yen)	76.8	73.5	69.6	65.6	62.3
	Mortality cost (billion yen)	1016.6	961.6	906.0	848.1	790.5
	[% of persons aged ≥ 65 years]	50.2%	49.2%	47.6%	46.0%	44.8%
	Mortality cost per person (million yen)	13.8	13.5	13.3	13.2	12.9
	COI (billion yen)	1597.6	1500.5	1400.7	1299.8	1204.1
Mixed model	Number of deaths (person)	73,775	71,088	68,120	64,106	60,992
	[% of persons aged ≥ 65 years]	87.8%	87.8%	87.6%	87.2%	86.9%
	Average age of death (year)	77.9	78.2	78.5	78.6	78.9
	Direct cost (billion yen)	530.5	491.3	457.5	419.7	380.0
	Morbidity cost (billion yen)	72.4	67.6	63.2	58.8	55.3
	Mortality cost (billion yen)	1016.1	960.2	903.5	844.3	785.2
	[% of persons aged ≥ 65 years]	50.2%	49.2%	47.7%	46.2%	45.1%
	Mortality cost per person (million yen)	13.8	13.5	13.3	13.2	12.9
	COI (billion yen)	1619.0	1519.1	1424.1	1322.8	1220.5

Source of estimated population: National Institute of Population and Social Security Research “Population Statistics of Japan”

#### Linear model and exponential/logarithmic model

The linear model indicated that COI tended to decrease from 2017 to 2026. It was predicted to decrease by 44.7% from 2014 to 2029, and the contribution rates of DC, MbC, and MtC were 30.4, 2.2, and 67.4%, respectively.

The linear model in the exponential/logarithmic projection indicated that COI tended to decrease from 2017 to 2026. It was predicted to decrease by 29.5% from 2014 to 2029, and the contribution rates of DC, MbC, and MtC were 49.1, 1.4, and 49.5%, respectively. Both types of projection predicted a decrease in NDy, DC, MbC, and MtC. All projected values were lower than those of the fixed model.

#### Mixed model

The mixed model was estimated by combinations of models of higher coefficients and was considered the most valid model in this study. This mixed model indicated that COI tended to decrease from 2017 to 2026. It was predicted to decrease by 28.6% from 2014 to 2029, and the contribution rates of DC, MbC, and MtC were 44.9, 2.9, and 52.3%, respectively. Regarding the calculation of DC from 2017 to 2029, THD and TOVy decreased, whereas iCd and oCd increased. The rates of change for THD, TOVy, iCd, and oCd were − 51.0, − 10.9, 149.5, and 103.9%, respectively. NDy was also predicted to decrease by 17.4% from 2014 to 2029, but no change was observed in the proportion of NDy of persons aged ≥ 65 years. The average age of death was 78.9 years in 2029, which slightly increased from 77.5 years in 2014. The proportion of MtC of persons aged ≥ 65 years was predicted to be 45.1% in 2029, and the MtC per capita was projected to decrease by 8.5% from 2014 to 2029.

#### Sensitivity analysis by discount rate

**Fig. 2:**
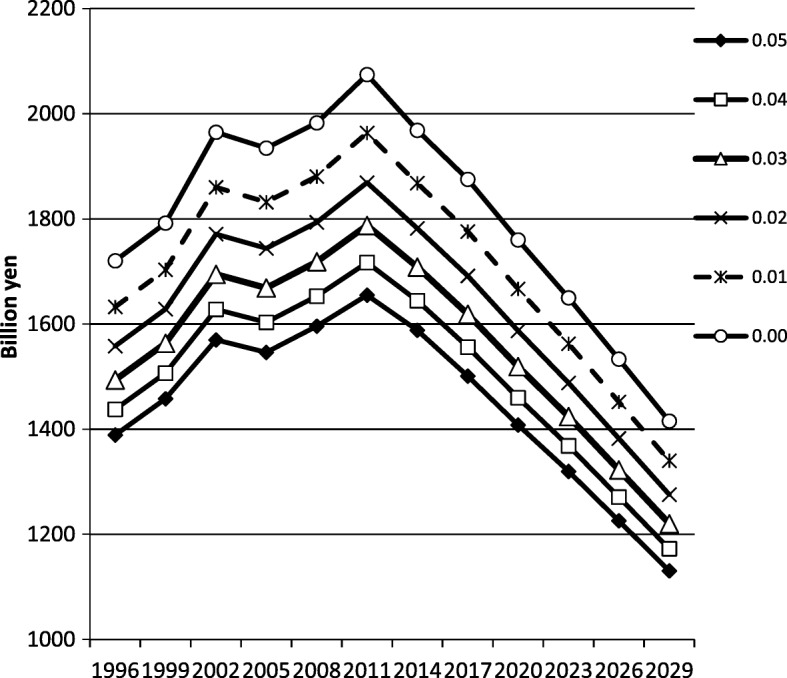
Sensitivity analysis discount rate varied from 0 to 5%; trend of COI remained the same

The results of the sensitivity analysis for the past estimation and mixed model projection are shown in Fig. 2. The change in the discount rate from 0 to 5% did not influence the trends observed in COI, and MtC remained the highest contributor to the COI.

## Discussion

The past estimation showed that a slight increase was found in the COI of IHD. DC had an unevenly increasing trend from 1996 to 2014. MbC showed decrease after peaking in 1999. MtC also had a tendency to increase until 2011, but decreased in 2014.

DC was influenced by THD, TOVy, iCd, and oCd. THD and TOVy decreased from 1996 to 2014. However, iCd and oCd remarkably increased, and the effect of this increase exceeded the effect of THD and TOVy decrease. The development of new therapies for IHD, such as drug and intra-vascular interventions, has been speculated to influence the increase in the unit cost.

MbC was calculated by multiplying TOVy/THD with LVd, and the main factor of the decrease of MbC was the decrease of ALOS. Recently, ALOS of all age groups have shown a remarkable decrease. Health policies encouraging shorter ALOS were introduced, including DPC/PDPS (Diagnosis Procedure Combination/Per-Diem Payment System), which is a reimbursement system introduced in 2003, where hospitals can receive more money with shorter length of stay, and admission criteria based on severity and needs for acute care hospitals in 2008 [27, 28].

MtC showed a tendency to increase. MtC was calculated by multiplying NDy with LVl (mean human capital value). In case of IHD, both NDy and LVl showed increasing tendencies until 2014. MtC was considered to be influenced by change in mortality rate, social aging (i.e., increasing number of elderly people), and change in LVl. At first, the reduction in mortality rate decreases NDy. Previously, a decrease in the mortality rate was observed in the elderly group (Table 1). If population structure, relative population of each age group, was stable, the NDy would decrease. In contrast, social aging has the effect of increasing overall NDy by increasing the population of elderly people with high mortality rate. Therefore, the increase in NDy in the past estimation has been speculated to occur because social aging effect exceeded the effect of the decrease in mortality rate. The increase in the average age of death by social aging also has the effect of reducing LVl at the time of death because the LVl of elderly people is low. In contrast, the increase in LVl of each sex and age group has the effect of increasing MtC. LVl showed remarkable increase in the elderly in particular from 1996 to 2014. Particularly, an increase of > 36.0% was found in the 70–74-year age group of both sexes, which was considered to be the result of the increase in labor force participation rate in the elderly and the increase in unpaid work, such as care for the spouse [21, 29]. The average age of death increased from 1996 to 2014, but the effect of reducing the LVl was speculated to be limited because the average age of death was already high in 1996, and the rate of increase was small. In other words, we can infer that the MtC increased because the effect of the increase in LVl in the elderly exceeded the effect of increase in the average age of death. Under the influence of these results, COI has been speculated to increase from 1996 to 2014.

Future projection of COI using the fixed model indicated that the COI of IHD would increase. However, if the trend of health-related indicators (mortality rate, per capita outpatient visits/hospitalizations, and ALOS) continues, COI will decrease in both linear and exponential/logarithmic models. The mixed model, which we consider to be the most credible model, also predicted that COI will show a decreasing tendency.

In the future, the proportion of the population in the ≥ 65 year age group will increase further, and NDy due to IHD will increase, but LVl at the time of death (value of human capital) will decrease because of the higher average age of death.

In the fixed model, NDy, DC, MbC, and MtC were predicted to increase. The effect of the decrease in per capita MtC was considered to offset the effect of the increase in NDy. In contrast, the mixed model showed that COI would decrease. As for the unit cost, the increase was estimated by the past trends; however, a remarkable decrease was predicted in THD and TOVy from 2017 to 2029. Therefore, it was estimated that DC will decrease. NDy and MtC decreased, the average age of death increased, and the per capita MtC decreased.

Previous studies have suggested that COI of major cancers decreased because of devaluation of human capital caused by the increase in the average age of death [13–17]. However, the average age of death by IHD was already high in 2014, and it is predicted to increase gradually. Therefore, COI of IHD was projected to be influenced mainly by the decrease in MtC due to the decrease in NDy. The effect of the decrease in the human capital value was expected to be small.

This study has several limitations. First, the COI method used here did not take the quality of the medical treatment provided or patients’ quality of life into consideration. Therefore, it does not examine the cost effectiveness of individual medical management. However, it is still useful because it enables future estimations and to regard the impacts of the aging population. Second, our model could not catch the effect of LVl increase on future MtC because our model fixed LVl in 2014. Our past estimation showed that LVl of the elderly was increasing, and it increased the MtC, which might underestimate our future projection.

However, projecting precise LVl per person classified by age groups is difficult. We cannot assume a permanent increase of labor force participation rate and unpaid work of the elderly, and these effects on future LVl are considered to be restrictive. In addition, the study period was relatively short, and dramatic changes occurred in the health care system during this period. However, the change among the different methods for determining projections was small, and therefore, the projections are likely to be accurate for the near future.

## Conclusion

The COI of IHD was estimated using Japanese government statistics. The past estimation showed that the COI of IHD increased. DC increased because of the increasing unit costs of developing new IHD therapies, MbC decreased because of the decrease in ALOS, and MtC increased because of social aging. In the mixed model, which we believe has the highest degree of relevance in future projection, the COI of IHD was predicted to decrease from 2017 to 2029 if the trend of health-related indicators (mortality rate, per capita outpatient visits/hospitalizations, and ALOS) continues. Possible factors contributing the change in the COI of IHD include aging of the population, DC, MtC, and NDy, and the impact of the aging of the population was considered the most significant.

## Abbreviations

ALOS: Average length of hospital stay
COI: Cost of illness
COI_0_: COI at the baseline year
COI_*t*_: COI at year *t*
${\mathrm{Cost}}_0^i$: DC, MbC, and MtC costs at the baseline year
${\mathrm{Cost}}_t^i$: DC, MbC, and MtC costs at year *t*
DC: Direct cost
HC: Hospitalization cost
HPy: Number of annual hospitalized patients
IC: Indirect cost
iCd: Inpatient cost per day
Iy: Annual income per person
LVd: One-day labor value per person
LVl: Lifetime labor value per person
MbC: Morbidity cost
MtC: Mortality cost
NDy: Number of deaths
OC: Outpatient cost
oCd: OC per day
THD: Total person-days of hospitalization
TOVy: Total person-days of outpatient visits
ULVy: Annual monetary valuation of unpaid work per person
